# Mechanisms and direction of allocation bias in randomised clinical trials

**DOI:** 10.1186/s12874-016-0235-y

**Published:** 2016-10-07

**Authors:** Asger Paludan-Müller, David Ruben Teindl Laursen, Asbjørn Hróbjartsson

**Affiliations:** 1 The Nordic Cochrane Centre, Rigshospitalet 7811, Copenhagen, Denmark; 2 Centre for Evidence-Based Medicine, University of Southern Denmark and Odense University Hospital, Odense, Denmark

**Keywords:** Allocation bias, Selection bias, Bias mechanisms, Systematic review, Randomised clinical trials, Direction of bias

## Abstract

**Background:**

Selective allocation of patients into the compared groups of a randomised trial may cause allocation bias, but the mechanisms behind the bias and its directionality are incompletely understood. We therefore analysed the mechanisms and directionality of allocation bias in randomised clinical trials.

**Methods:**

Two systematic reviews and a theoretical analysis. We conducted one systematic review of empirical studies of motives/methods for deciphering patient allocation sequences; and another review of methods publications commenting on allocation bias. We theoretically analysed the mechanisms of allocation bias and hypothesised which main factors predicts its direction.

**Results:**

Three empirical studies addressed motives/methods for deciphering allocation sequences. Main motives included ensuring best care for patients and ensuring best outcome for the trial. Main methods included various manipulations with randomisation envelopes. Out of 57 methods publications 11 (19 %) mentioned explicitly that allocation bias can go in either direction. We hypothesised that the direction of allocation bias is mainly decided by the interaction between the patient allocators’ motives and treatment preference.

**Conclusion:**

Inadequate allocation concealment may exaggerate treatment effects in some trials while underestimate effects in others. Our hypothesis provides a theoretical overview of the main factors responsible for the direction of allocation bias.

**Electronic supplementary material:**

The online version of this article (doi:10.1186/s12874-016-0235-y) contains supplementary material, which is available to authorized users.

## Background

In 1948 the British Medical Journal published the UK Medical Research Council’s famous trial of streptomycin treatment for pulmonary tuberculosis which is generally regarded the first properly conducted randomised clinical trial [1].

The planners of the streptomycin trial conceptualised randomisation as a two-step process. The first step was the generation of a random sequence: “*… random sampling numbers [were] drawn up for each sex at each centre by Professor Bradford Hill*” [1]. The second step was the concealment of the random sequence from the person responsible for inclusion of patients: *“… the details of the series … were contained in a set of sealed envelopes, each bearing on the outside only the name of the hospital and a number.”* In modern terminology these two components of randomisation are usually called “allocation sequence generation” and “allocation sequence concealment”.

The main methodological rationale for randomisation in a clinical trial is to protect against allocation bias, i.e. to facilitate equal baseline distribution of prognostic factors between the compared groups. Allocation bias may result from investigators knowing or predicting which intervention the next eligible patient is supposed to get. This knowledge may influence the information provided to patients or the threshold for approaching a potentially eligible patient, thereby selecting patients with a good prognosis (i.e. anticipated good outcome and treatment response) into one of the groups. Concealment of the allocation sequence is often regarded the most important aspect of the randomisation process.

A trial is very vulnerable to allocation bias if no attempts have been made to conceal the allocation sequence (for example the allocation list is openly available). A more frequent scenario consists in inadequate concealment methods enabling deciphering of allocation sequence (for example opening unsealed and unnumbered convolutes) or intelligent guessing of allocations when restricted randomisation is used (for example block randomisation). In the latter case, trials that use small, fixed and openly available block sizes may enable intelligent guessing of a considerable proportion of patients (for example if block size is 2, the allocated intervention can be deduced for half of the patients).

The theoretical considerations on allocation bias found considerable support in a pivotal study of allocation concealment (and other aspects of trial design), published in 1995 by Schulz and colleagues [2]. They reported that, on average, trials with inadequate allocation concealment exaggerated estimated treatment effects, i.e. odds ratios, by 41 %. This dramatic result promoted inadequate allocation concealment as a leading cause of bias in clinical trials. For example, up to 2008 allocation concealment was the only risk of bias item incorporated in RevMan, the software used for Cochrane Reviews [3]. However, the result also encouraged a conception that allocation bias - to a considerable extent - was unidirectional, typically exaggerating the experimental intervention.

Since 1995 several meta-epidemiological studies of allocation concealment have reported inconsistent results. An overview from 2012 combined seven such studies and reported that trials with unclear or inadequate allocation concealment exaggerated treatment effects by only 7 % [4] and that the degree of bias, as well as the degree of variation in estimated treatment effect from trial to trial, was more pronounced in trials with subjective outcomes. Another overview from 2016 found similar results [5]. Thus, the magnitude of allocation bias appears considerably more uncertain than previously believed and related to background factors, such as type of outcome.

Already in 1995 Schulz and colleagues emphasised that allocation bias may *“inconsistently bounce in both directions …”* [2]*.* However, this observation had much less impact than their estimate of an average degree of bias. If allocation bias indeed “bounce in both directions” in more than a rare minority of trials it becomes a relevant question which factors cause the directionality. We have not identified any comprehensive analysis of this question.

Thus, we decided to study the mechanisms and directionality of allocation bias. Our main objectives were: I) to systematically review empirical studies of the motives and methods of investigators allocating patients in a trial with no, or inadequate, allocation concealment, or trials where upcoming allocations are predictable, despite of allocation concealment; II) to systematically review methods publications commenting on mechanisms and directionality of allocation bias; and III) to suggest a reasoned hypothesis for which main factors decide the direction of allocation bias.

## Methods

This study consists of two systematic reviews and a theoretical analysis.

### General terminology

By “allocation bias” we understand the bias caused by allocating patients with better prognosis to either the experimental or the control group. In the context of a randomized trial the term “selection bias” is sometimes used instead of allocation bias to indicate selection of patients into treatment arms. We avoid the term “selection bias” as it has a different meaning in epidemiology more broadly: selection of non-representative persons into a study.

Concealment of allocation is sometimes confused with blinding. We adhere to the standard terminology in which “concealment of allocation” takes place before and during patient allocation, whereas “blinding” (i.e. keeping allocations hidden from trial participants, for example patients and investigators) occurs after patient allocation. We define adequate concealment of allocation as not only thwarting knowledge of future allocations, but also prediction due to intelligent guessing, for example in trials with block randomisation.

We call the person in a randomised clinical trial responsible for recruitment and allocation of patients for the “patient allocator”.

### Systematic review of studies of the motives and behaviours of patient allocators

We identified, summarised and analysed empirical studies of the motives and behaviours of investigators participating in a trial with no allocation concealment or deciphering the allocation sequence in a trial with intended allocation concealment. We included all types of empirical studies examining patient allocator motives and behaviour in more than a single trial, for example questionnaire surveys, interview studies, or personal accounts. We excluded studies based only on a single trial (but noted their results, and used these for a sensitivity analysis).

We searched PubMed, the Cochrane Methodology Register, and Google Scholar (see Additional file 1 for detailed search strategy), and systematically read the list of references in eligible studies and review articles. The search was conducted on the 15^th^ of March 2015. No language restrictions were applied. One author (AP) screened all hits. If a text was judged potentially eligible, the full text was read, and if clearly ineligible excluded. All other publications were read by two authors (AP and AH or DL).

Two authors (AP and either DL or AH) extracted data independently. Disagreement was solved by discussion. We extracted: type of study (i.e. questionnaire or interview, vignette or actual trial), characteristics of the patient allocator, mechanism used to decipher allocation sequence, and motives involved. We contacted the authors of eligible studies if we thought that more detailed outcome data could be obtained.

The design and results of each individual study were noted. Risk of bias was assessed based on the individual study design, i.e. in a questionnaire study mainly the response rate and the risk of response bias and in a study based on recollection of conversations with course participants mainly the risk of response bias. The results of the studies were summarised qualitatively.

As a sensitivity analysis we summarised studies of allocator motives and behaviour when based only on a single trial.

### Systematic review of methods publications

We identified, summarised and analysed methods publications that commented on mechanisms or direction of allocation bias. We included all types of publications, for example textbooks on trial methodology and clinical epidemiology and relevant sections from meta-epidemiological studies, articles, editorials etc. We focused on identifying publications with a primary objective to discuss allocation bias, but did also include publications that commented on allocation bias without this being the primary objective. We excluded publications that did not comment on either mechanisms or directionality of allocation bias or that only expressed peripheral remarks, i.e. remarks that were short (for example 1–2 lines) or tangential to the subject (for example allocation bias was mentioned but not reflected upon).

We searched PubMed, the Cochrane Methodology Register, Google Scholar, and selected textbooks using the search strategy developed by Moustgaard and colleagues [5] and systematically read the references to eligible studies and review articles (Additional file 1). The data-base search was conducted on the 17^th^ of March 2015. No language restrictions were applied.

One author (AP) screened all hits. If a text was judged potentially eligible, the full text was read, and if clearly ineligible excluded. All other publications were read by two authors (AP and AH or DL). Disagreement was solved by discussion.

Two authors (AP and DL) extracted data independently. Disagreement was solved by discussion.

For any included methods publication we extracted the following data: type of publication (theoretical article, meta-epidemiological study, textbook, comment or editorial), publication year, main subject (allocation concealment/allocation bias or other), and the verbatim quote of any comment on the mechanisms or direction of allocation bias.

The individual comments were categorised according to main content and tabulated based on general characteristics, direction of allocation bias and mechanisms of allocation bias.

### Hypothesis for the main factors deciding the direction of allocation bias

We developed a reasoned methodological hypothesis (i.e. small theoretical model) for how motives and treatment preferences in patient allocators transform into a biased result in at trial. Based on methodological theory (partly summarised in the previous parts of our paper) as well as empirical studies [4, 5], we defined the main motives and treatment preferences of the patient allocator in a clinical trial, and analysed how this could influence the allocation of patients and the direction of allocation bias. In formulating a hypothesis we aimed for one that that was coherent with the main empirical studies, logically consistent, and intuitively plausible. We also restricted our focus to the classical situation of a two group parallel group randomised trial aiming to study whether the experimental intervention was superior to the control intervention.

By direction of bias we mean whether the outcome of a trial exaggerates or underestimates the true treatment effect. Thus, we use the term to determine the bias on a trial level (not on the level of the individual allocator, or the level of a meta-analysis).

## Results

### Systematic review of studies of the motives and behaviours of patient allocators

Based on screening of 3419 publications, we read the full text of 43 and excluded 40 (Additional file 1). Thus, we included three studies of patient allocators’ behaviour and motives when either participating in a trial without allocation concealment or deciphering the allocation sequence in a trial intending to conceal the sequence [6–8].

Hewitt 2009 was a web-based survey of investigators who were, or had been, involved in clinical trials [6]. A total of 125 trial investigators were identified through personal contacts and mailing lists of trial relevant organisations in the UK, 15 additional groups were identified through the National Academic Mailing List service, and an unknown number of contacts made as invited participants were asked to forward the email to anyone they thought would be interested. A total of 268 persons responded to at least one question. The persons were asked how many trials they knew of in which randomisation had been subverted (i.e. deciphered), and were asked of an account of the separate incidences, including the reasons for deciphering.

Some 30 survey responders provided details of deciphering (numbers not reported) or involvement in trials with no concealment of allocation (numbers not reported). Of the 30 responders, 14 referred to personal experience, 11 referred to what others had told them, two referred to secondary analyses (it is unclear what “secondary analyses” means in this context) and one referred to the published literature (two responders did not clarify what was the basis for their example of deciphering). The deciphering methods involved are summarised in Table 1.

**Table 1:** Methods for deciphering ^a^ allocation sequence or otherwise tampering with allocations in randomized trials, based on a systematic review of three empirical studies on the motives and behaviors of investigators recruiting patients to clinical trials

Envelopes/drug containers
• Holding envelopes to light
• Opening envelopes before entering patient
• Entering two patients at the same time, and switching envelopes
• Judging weight difference between envelopes
• When using sequentially numbered drug containers, difference of appearance
Central randomisation
• Several allocations given from central office, at the same time
• Clinician informed of next allocation, before deciding whether to enter the patient
• Manipulation of lists (e.g. not writing patients on lists in the right order)
Others
• Prediction of future assignments based on past assignments, when using restricted randomisation (for example by keeping a log)
• Finding assignment sequence in chief investigators office

^a^ *Some studies employ no allocation concealment, in such cases no deciphering takes place, but the resulting bias remains the same*

A “frequently reported reason” for deciphering the allocation sequence was the intention to act in the interest of the participants, i.e. allocating patients in greater need to the treatment assumed to be best. A second “major reason” was showing that a particular treatment worked, i.e. facilitating a trial result that reflected the patient allocator’s preferred treatment. Other motives reported were: pressure from patients, pressure from others involved in the trial, practical/technical concerns, and lack of knowledge. The survey did not report in detail which motive was the most frequent. Contact to the authors revealed that the study data were not available.

Schulz 1995 summarised conversations with methodological workshop participants (over 20 workshops with 20–25 persons each) conducted from 1988 to 1995 [7]. About half of the >400 participants recalled, or had witnessed, a case of deciphering allocation sequence. There were no explicit accounts of participants being part of trials without any allocation concealment. The deciphering methods involved are summarised in Table 1.

Schulz and colleagues did not ask about the motives for deciphering, but they got some volunteered comments. Frequently, workshop participants simply lacked knowledge of the scientific ramifications while in one case a surgeon wanted experience in a specific operating procedure.

Brown [8] asked 25 clinicians and research nurses who were actively recruiting patient into trials run by a clinical trials unit whether they tried to predict patient allocations, and if so, their motives and methods. Four of the 25 (16 %) stated that they tried to predict future allocations. The motives reported by “a few” were that they hoped that certain patients would be allocated to a specific treatment, giving reasons such as “some would have a perceived benefit from a particular treatment” or “patients would be able to travel more easily”. The method used was by keeping a log of all previous patient allocations (Table 1).

We considered the risk of bias as notable in all three studies. Hewitt 2009 could not account the number of potential participants in their survey due to their study design so the response rate was not possible to assess. Schulz 1995 involved recollections of informal conversations in public, so there is a considerable risk of response bias. Furthermore, both studies included cases of what others had witnessed (and in Hewitt 2009 one case based on a published account). Brown 2005 was an explorative study with a small sample size, and it was unclear whether the answers were the result of a written questionnaire or an oral interview.

As a sensitivity analysis we noted any reports of motives or methods of deciphering allocation sequence based on a singular trial (which were excluded from the main review). We identified five such trials. In one trial the motives for deciphering was interpreted to be practical considerations (but this was not based on direct interviews with the involved patient allocators). In the remaining four trials the mechanisms mentioned either involved manipulations with envelope randomisation or in one trial that the coordinating investigator knew the allocation schedule (Additional file 1).

### Systematic review of methods publications

Based on screening of 1852 publications, we read the full text of 130 publications of which 73 were excluded (Additional file 1). Thus, we included 57 publications [2, 4, 6, 9–62], (Tables 2 and 3).

**Table 2:** Characteristics of methods publications addressing allocation bias directionality and mechanisms

Publication characteristics	n (%)
Total	57
Publication type	
Theoretical article	33 (58 %)
Meta-epidemiological study	9 (16 %)
Textbook	12 (21 %)
Editorial	1 (2 %)
Comment	2 (3 %)
Publication topic	
Allocation bias primary aim	32 (56 %)
Other	25 (44 %)
Year of Publication	
1975–1984	1 (2 %)
1985–1994	3 (5 %)
1995–2004	18 (32 %)
2005–2014	35 (61 %)

**Table 3:** Major points in 57 methods publications addressing allocation bias directionality and mechanisms

Direction of bias	N^a^ (%)
Studies explicitly commenting on direction of bias	27 of 57 (47 %)
Studies commenting only on exaggeration of treatment effect	16 (28 %)
Studies commenting on possible bias in both directions	11 (19 %)
Studies not explicitly commenting on direction of bias	30 of 57 (53)
Mechanisms of bias	
Allocation concealment protects against allocation bias	53 of 57 (93 %)
Preferential allocation of patients may lead to bias.	32 of 57 (56 %)
Allocation bias is possible in trials with restricted randomisation^b^, if upcoming assignments can be guessed.	11 of 57 (19 %)

^a^Number; ^b^restricted randomisation refers to the process of making restrictions to the randomisation scheme (e.g. blocked randomisation)

The directionality of allocation bias, was explicitly commented on in 27 of 57 publications (53 %), however only 11 (19 %) [2, 14, 15, 29, 30, 42, 48, 51, 59, 61, 62] pointed out that allocation bias can be bidirectional, the remaining 16 (28 %) [9, 13, 18–20, 26–28, 31, 34, 37, 38, 46, 53–55] only mentioned that treatment effects could be exaggerated.

Eight of the 57 publications (14 %) [21, 22, 28, 30, 32, 42, 44, 61] proposed hypothetical scenarios in which motives of patient allocator result in potential allocation bias (Table 4).

**Table 4:** Hypothetical scenarios described in 57 methods publications addressing allocation bias directionality and mechanisms

Favouring treatment:	Favouring control:	Direction unpredictable:
*“An investigator for a pharmeceutical company, very anxious to see the company’s latest pharmaceutical product succeed, guesses the randomization sequence and randomizes patients he or she deems more likely to respond positively to the new therapy when he believes the new therapy to be next in the sequence”* [21]^*a*^ *“Or older patients might receive the traditional therapy and youngsters the new one”* [41] *“Because of the vested interest the sponsor may have in the outcome of the trial (Hogel and Gaus, 1999), there may be a temptation to attempt to bias trials towards more favorable outcomes.“* [27] *“…when clinicians know ahead of time which treatment their next eligible and consenting patient will receive, they may (consciously or unconsciously) enter patients with lower risk and/or higher responsiveness into the experimental treatment group. As a result, the trial can become biased in favor of experimental therapy from the start.”* [29]	*“The clinician might, for example, allocate the patients with the worst prognosis to the, in his/her opinion, ‘promising’ new therapy and the better ones to the older treatment, no doubt with the best possible intention in respect of his/her patients”* [41] *“On the other hand, in some unblinded trials, one of the treatments may be systematically assigned to the sickest patients. Randomization appears to have been systematically slanted to make these patients benefit from the test medication which would appear to be more effective.”* [31]	*“A sympathetic nurse coordinator tries to assign a favorite patient to the new therapy rather than placebo”* [21] *“If the referring health care provider knows the next subject will be allocated to Slimmenow* ^*b*^ *, he/she may be inclined to try to help a certain patient he/she thinks may benefit more. Or perhaps knowing the next subject is to be allocated to placebo, he/she refers someone who really does not need to lose much weight.”* [61] *“They perhaps “know” the more effective treatment, so they may want certain patients to benefit or may want the results of a study to reveal what they believe to be valid.”* [20] *“For instance, if the referring clinician thinks that treatment A is less effective than treatment B, and he/she knows that the next subjects will be allocated to treatment A and B, respectively, he/she may be inclined to chose a patient with mild symptoms for treatment A, and a patient with more severe symptoms for treatment B.”* [43]

^a^References indicate from which publication scenario is taken; ^b^Hypothetical experimental treatment

Had we included only the 32 publications with a main focus of addressing allocation bias, the results would have been similar: 18 of 32 (56 %) publications commented on direction of bias, of which 6 (19 %) pointed out that bias can be bidirectional.

### Hypothesis for the main factors deciding the direction of allocation bias

**Fig. 1:**
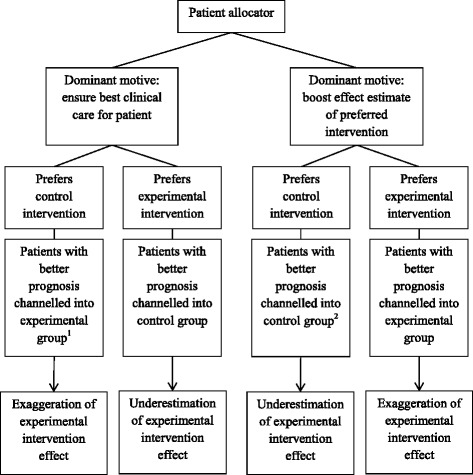
The preference-motive interaction hypothesis for direction of allocation bias in randomised clinical trials. ^1^If dominant motive is best care for patient, we expect patient allocator to channel patients with the worst prognosis into his/her preferred group, as these patients need the most effective treatment the most; ^2^If the dominant motive is to boost the effect estimate of the preferred intervention, we expect patient allocator to channel patients with better prognosis into preferred group, so the intervention will look more effective

We developed a reasoned methodological hypothesis (i.e. small theoretical model) for the direction of bias in a parallel group superiority randomised trial. We assumed that the main factors involved were patient allocators’ treatment preference (either the experimental intervention or the control intervention) and their motive (either securing best patient care or boosting trial result). By analysing how these factors could interact we derived at a model, summarised in Fig. 1 and developed in more detail below.

In a clinical trial a patient allocator will typically prefer either the experimental or the control intervention. The third possible situation involves a patient allocator who is fully and truly neutral in all relevant clinical situations (in which allocation bias is not a problem and concealment of allocation unnecessary); however, this is a very unlikely situation (even patient allocators who consider themselves neutral may not be so).

Which intervention is preferred (and the degree of preference) differs from person to person and in a single trial all patient allocators may not agree. Still, the initiation of a randomised trial is typically driven by a considerable degree of hope and positive expectations, so seen as a group the patient allocators may typically prefer the novel experimental intervention.

However, in special situations the patient allocators may prefer the control intervention: when the effect of the experimental intervention is regarded negligible or inferior, for example in a trial of homeopathy vs. standard care for cancer, in an experimental non-surgical intervention vs. a control routine surgical intervention, or in a trial of the experimental oral format of a drug vs. its intravenous control format [63].

Furthermore, the patient allocator typically has two conflicting main motives: best patient care and best possible trial results. Which motive is the dominant one will affect the direction of allocation bias. A patient allocator who wants to (consciously or unconsciously) boost the estimated effect of the preferred intervention will need to channel patients with a good prognosis into the preferred intervention arm. On the contrary, a patient allocator who wants the best care for patients will need to channel patients with bad prognosis to the intervention perceived as more effective as patients with a bad prognosis are those in greatest need of the best treatment. The motives may differ between patient allocators in a given trial, but the net impact on a trial will reflect the motives of the patient allocators as a group.

This hypothesis presupposes the ability of the patient allocator to (better than chance) predict the prognosis of patients, for example based on age, disease stage and co-morbidity [64, 65]. Such prognostic ability may differ according to type of disease or type of outcome. However, our hypothesis presupposes that differences in prognostic ability do not impact on the direction of allocation bias, only on its degree. For simplicity we have not incorporated patient preferences into the hypothesis.

## Discussion

Based on the three empirical studies, the main motives for influencing patient allocation in a randomised trial seem to be boosting the estimated effect of the experimental intervention and to ensure best care for the patient. Of 57 methods publications commenting on allocation bias 11 (19 %) explicitly stated its dual directionality. We developed a hypothesis that the direction of allocation bias is mainly influenced by the interaction between the patient allocators’ treatment preference (experimental or control) and dominant motive (best patient care or boosting trial results).

To our knowledge this is the first review of studies of the mechanisms and directionality of allocation bias. Only three eligible studies were identified. An older study from 1995 was based on recollections of what >400 participants in methodology workshops had revealed. Their experience may not be representative of contemporary trials and recollections may not be fully reliable. A study from 2005 was a small study with only 25 participants. The newer study from 2009 was a web-based survey of >268 participants. Only 30 responders provided details of mechanisms and motives of deciphering. Results from the three studies were consistent, but clearly there is a high risk of both non-response bias [66] and response bias [67] in such surveys of problematic behaviour. We stress that our results reflect reported conduct, which may not accurately reflect actual conduct. There remains a fundamental uncertainty of how often deciphering takes place, but little doubt that it does take place, and it is likely that the motives for deciphering allocation sequence differ considerably.

We are fairly confident that we identified most methods publications aiming to discuss allocation bias. However, minor comments in publications with other primary intentions are challenging to identify and some may have been overlooked. Also, the distinction between minor comments (which are included) and peripheral statements (which are excluded) is to some degree subjective. Our results, however, were similar when excluding minor comments. Our hypothesis proposes a simple mechanism for the dual directionality of allocation bias, and was informed by empirical studies and methods publications.

Considering the importance of allocation bias for trial methodology it is remarkable that only three studies of the mechanism and motives for allocation deciphering seem to have been conducted. We identified five trials reporting on potential deciphering in individual trials, all had been identified and commented on before [29]. We excluded such studies because of a high risk of publication bias. Still, the results of these trials were consistent with that of the studies we included. This lack of research into mechanisms and motives may partly reflect a prevalent conception among researchers that empirical studies of allocation bias are more important than studies of its mechanisms, or that the challenges involved in asking trial investigators about potentially problematic procedures might be too difficult to overcome.

The problem of the directionality of allocation bias is part of a broader discussion of the direction of bias in general. Kunz and colleagues compared randomised and non-randomised studies and reported a heterogeneous pattern of results without one design type, on average, finding higher estimates of treatment effects. They coined the phrase “unpredictability paradox” to describe this phenomenon [14]. Though it is unclear to which degree such studies compare “like with like”, the underlying predicament could well be the directionality of bias. A similar problem could impact on the empirical challenges of assessing other types of bias in randomised trials, for example bias due to attrition, blinding and outcome selection [5, 68].

A main theoretical discussion following Schulz and colleagues’ 1995 study [2] was whether the association between inadequate allocation concealment and exaggerated treatment effects was causal or whether it (to some extent) was a marker of other biases. The finding in meta-epidemiological studies that bias was more pronounced in trials with subjective outcomes was unexpected [4, 5]. One possibility is that allocation concealment and blinding may have been incompletely separated in the meta-epidemiological studies, for example due to unclear reporting in trial reports. Another possibility is that clinicians are less able to predict prognosis in trials that assess mortality (for example in large preventive cardiovascular trials) than in other trials. Both interpretations fit with the mechanisms of our proposed hypothesis.

Which patient allocator motive is the dominant one in a trial differs from patient allocator to patient allocator. It is interesting to reflect on possible factors associated with such motives. Boosting the estimated treatment effect may be a dominant motive in trials where the patient allocator is deeply involved in the planning, design and execution of the trial (or if an allegiance has been established between the investigators and the sponsors of the trial) and in trials including patients with a reversible condition, for example acute pain. Similarly, best patient care may be a more dominant motive in trials in which the patient allocator’s only role is including patients or in trials including patients with a potentially irreversible condition (high risk of death or of permanent disability).

Our hypothesis thus predicts that allocation bias in many trials initiated and run by clinicians will exaggerate the estimated treatment effect when the patient allocators prefer the experimental intervention. This is probably a fairly typical situation, also because inadequate allocation concealment tends to be more frequent in such trials. Many contemporary trials initiated and run by other than the investigator (for example a foundation, research council or industry) will be large multi-centre trials. Such trials are often conducted with concealment of allocation, but our hypothesis predicts that if clinicians consider the experimental intervention superior, any allocation bias may tend to underestimate the experimental treatment effect. However, the direction of bias may partly depend on the ability of the initiators of the trial, for example a drug company, to create a bond of loyalty in the investigators who allocate patients. Thus, though we hypothesise the main factors behind the directionality of allocation bias, it remains challenging to predict the direction of allocation bias in an individual trial without knowledge of the preferences and motives of the involved clinicians.

Our hypothesis was not designed to be tested empirically, and there are considerable difficulties in studying the relation between patient allocators’ motives, treatment preferences and actual behaviour in a real trial, partly because of a high risk that the initiation of the study would change the behaviour it is set up to assess. A practically feasible but more indirect study approach would be to ask actual or possible patient allocators about treatment preferences, motives and behaviour based on constructed theoretical scenarios.

Allocation bias is driven by the lack of neutrality of the clinicians including patients into the trial. This contrasts the ethical prerequisite for a clinical trial: equipoise, i.e. that there is a considerable degree of uncertainty with respect to which intervention is best. From an academic perspective, equipoise is an attractive ideal, but in practice there will be a considerable difference between the position of individual clinicians and the clinical community at large [69], and also a large grey zone in which some uncertainty coexists with a considerable degree of expectation that an experimental intervention is effective.

Lack of insight in the scientific importance of allocation bias is probably an important background factor for allocation bias but ignorance is insufficient in itself for inducing allocation bias. If all persons involved in the unconcealed allocation process in a trial were ignorant about the scientific importance of avoiding allocation bias and had no preferences errors in the allocation process would occur by chance, which would increase random variation but not cause bias.

Our model presupposes a parallel group randomized trial. In cross-over trials allocation bias will have less influence on treatment estimate effects as patients will receive both experimental and control intervention during the trial. Our model also presupposes a superiority approach in which the purpose of the trial is to test whether the experimental intervention is superior to the control intervention. Some trials are designed as non-inferiority trials. Which intervention is preferred by the person responsible for patient allocation is less clear in such trials. In some situations the experimental intervention is preferred because it may be more practical to administer but in other trials it may be considered inferior to the established control intervention [63].

Our study provides a framework for the interpretation of risk of bias in clinical trials with inadequate allocation concealment (for example when included in meta-analyses) and for the interpretation of results of meta-epidemiological studies. Our study also documents a paucity of empirical studies on the treatment preferences and motives of investigators allocating patients in clinical trials. Considering the importance of allocation bias to clinical trials methodology, we suggest that further studies of the mechanisms of bias are warranted. Awaiting results from such studies, our hypothesis can be used as a tentative conceptual tool when reflecting on the direction of allocation bias in a given trial.

## Conclusion

Trial investigators who allocate patients in a randomised trial may have different treatment preferences and conflicting motives for influencing patient allocation. Inadequate allocation concealment may exaggerate treatment effects in some trials while underestimate effects in others. This important dual directionality of allocation bias is not a prominent feature of trial methodology publications. It is challenging to predict the direction of allocation bias in an individual trial without knowledge of the preferences and motives of the involved clinicians, but our hypothesis provides a theoretical overview of the main factors responsible for the direction of allocation bias.

## Additional file

Additional file 1: Search Methods and Sensitivity Analysis.(DOCX 44 kb)

## References

[1] Streptomycin Treatment of Pulmonary Tuberculosis. Br Med J. 1948 Oct 30;2(4582):769–82. PMC2091872 18890300

[2] Schulz KF, Chalmers I, Hayes RJ, Altman DG (1995). Empirical evidence of bias. Dimensions of methodological quality associated with estimates of treatment effects in controlled trials. JAMA.

[3] Hróbjartsson A, Boutron I, Turner L, Altman DG, Moher D (2013). Assessing risk of bias in randomised clinical trials included in Cochrane reviews: the why is easy, the how is a challenge. Cochrane Database Syst Rev.

[4] Savović J, Jones HE, Altman DG, Harris RJ, Jüni P, Pildal J (2012). Influence of reported study design characteristics on intervention effect estimates from randomized, controlled trials. Ann Intern Med.

[5] Page MJ, Higgins JPT, Clayton G, Sterne JAC, Hróbjartsson A, Savović J (2016). Empirical evidence of study design biases in randomized trials: systematic review of meta-epidemiological studies. PLoS One.

[6] Hewitt CE, Torgerson DJ, Berger VW (2009). Potential for technical errors and subverted allocation can be reduced if certain guidelines are followed: examples from a web-based survey. J Clin Epidemiol.

[7] Schulz KF (1995). Subverting randomization in controlled trials. JAMA.

[8] Brown S, Thorpe H, Hawkins K, Brown J (2005). Minimization--reducing predictability for multi-centre trials whilst retaining balance within centre. Stat Med.

[9] Chalmers TC, Celano P, Sacks HS, Smith H (1983). Bias in treatment assignment in controlled clinical trials. N Engl J Med.

[10] Grimes DA (1991). Randomized controlled trials: “it ain’t necessarily so”. Obstet Gynecol.

[11] Schulz KF, Chalmers I, Grimes DA, Altman DG (1994). Assessing the quality of randomization from reports of controlled trials published in obstetrics and gynecology journals. JAMA.

[12] Schulz KF (1995). Unbiased research and the human spirit: the challenges of randomized controlled trials. CMAJ Can Med Assoc J.

[13] Schulz KF (1996). Randomised trials, human nature, and reporting guidelines. Lancet Lond Engl.

[14] Kunz R, Oxman AD (1998). The unpredictability paradox: review of empirical comparisons of randomised and non-randomised clinical trials. BMJ.

[15] Berger VW, Exner DV (1999). Detecting selection bias in randomized clinical trials. Control Clin Trials.

[16] Altman DG, Bland JM (1999). Statistics notes. Treatment allocation in controlled trials: why randomise?. BMJ.

[17] Torgerson DJ, Roberts C (1999). Understanding controlled trials. Randomisation methods: concealment. BMJ.

[18] Schulz KF (2001). Assessing allocation concealment and blinding in randomised controlled trials: why bother?. Evid Based Nurs.

[19] Swingler GH, Zwarenstein M (2000). An effectiveness trial of a diagnostic test in a busy outpatients department in a developing country: issues around allocation concealment and envelope randomization. J Clin Epidemiol.

[20] Jüni P, Altman DG, Egger M (2001). Systematic reviews in health care: assessing the quality of controlled clinical trials. BMJ.

[21] Altman DG, Schulz KF (2001). Statistics notes: concealing treatment allocation in randomised trials. BMJ.

[22] Rosenberger, W. F. and Lachin, J. M. (2002) Selection Bias, in Randomization in Clinical Trials: Theory and Practice, John Wiley & Sons, Inc., Hoboken, NJ, USA. doi:10.1002/0471722103.ch6.

[23] Beller EM, Gebski V, Keech AC (2002). Randomisation in clinical trials. Med J Aust.

[24] Schulz KF, Grimes DA (2002). Unequal group sizes in randomised trials: guarding against guessing. Lancet Lond Engl.

[25] Berger, Vance W. and Christophi, Costas A. Randomization technique, allocation concealment, masking, and susceptibility of trials to selection bias. J Mod Appl Stat Methods. 2003;2(1):8.

[26] Berger VW, Weinstein S (2004). Ensuring the comparability of comparison groups: is randomization enough?. Control Clin Trials.

[27] Pildal J, Chan A-W, Hróbjartsson A, Forfang E, Altman DG, Gøtzsche PC (2005). Comparison of descriptions of allocation concealment in trial protocols and the published reports: cohort study. BMJ.

[28] Berger VW (2005). Quantifying the magnitude of baseline covariate imbalances resulting from selection bias in randomized clinical trials. Biom J Biom Z.

[29] Berger V (2005). Selection bias and covariate imbalances in randomzied clinical trials.

[30] Haynes RB, Sackett DL, Guyatt GH, Tugwell P. Clinical Epidemiology. How to do clinical practice research, 3rd ed. Philadelphia: Lippincott Williams and Wilkins; 2005.

[31] Doig GS, Simpson F (2005). Randomization and allocation concealment: a practical guide for researchers. J Crit Care.

[32] Spriet A, Dupin-Spriet T (2005). Good practice of clinical drug trials.

[33] Hewitt C, Hahn S, Torgerson DJ, Watson J, Bland JM (2005). Adequacy and reporting of allocation concealment: review of recent trials published in four general medical journals. BMJ.

[34] Scales DC, Adhikari NKJ (2005). Maintaining allocation concealment: following your SNOSE. J Crit Care.

[35] Forder PM, Gebski VJ, Keech AC (2005). Allocation concealment and blinding: when ignorance is bliss. Med J Aust.

[36] Akobeng AK (2005). Understanding randomised controlled trials. Arch Dis Child.

[37] Attia AM (2005). Bias in RCTs: confounders, selection bias and allocation concealment. Middle East Fertil Soc J.

[38] Gluud LL (2006). Bias in clinical intervention research. Am J Epidemiol.

[39] Machin D, Day S, Green S. Textbook of Clinical Trials. 2nd ed. Chichester: John Wiley & Sons, Ltd; 2006. doi:10.1002/9780470010167.

[40] Pildal J, Hróbjartsson A, Jørgensen KJ, Hilden J, Altman DG, Gøtzsche PC (2007). Impact of allocation concealment on conclusions drawn from meta-analyses of randomized trials. Int J Epidemiol.

[41] Levin KA (2007). Study design VII. Randomised controlled trials. Evid Based Dent.

[42] Everitt B, Wessely S. Clinical trials in psychiatry. Chichester, England; Hoboken, NJ. John Wiley & Sons; 2008.

[43] Wood L, Egger M, Gluud LL, Schulz KF, Jüni P, Altman DG (2008). Empirical evidence of bias in treatment effect estimates in controlled trials with different interventions and outcomes: meta-epidemiological study. BMJ.

[44] Cipriani A, Nosè M, Barbui C (2008). Allocation concealment and blinding in clinical trials. Epidemiol Psichiatr Soc.

[45] Hackshaw A. Front Matter, in A Concise Guide to Clinical Trials. Oxford: Wiley-Blackwell; 2009. doi:10.1002/9781444311723.

[46] Nüesch E, Reichenbach S, Trelle S, Rutjes AWS, Liewald K, Sterchi R (2009). The importance of allocation concealment and patient blinding in osteoarthritis trials: a meta-epidemiologic study. Arthritis Rheum.

[47] Grobbee DE, Hoes AW (2009). Clinical epidemiology: principles, methods, and applications for clinical research.

[48] Friedman LM, Furberg C, DeMets DL. Fundamentals of clinical trials. New York: Springer; 2010.

[49] Machin, D. and Fayers, P. M. (2010). Randomized Clinical Trials: Design, Practice and Reporting, John Wiley & Sons, Inc., Hoboken, NJ, USA. doi:10.1002/9780470686232.

[50] Kennes LN, Cramer E, Hilgers R-D, Heussen N (2011). The impact of selection bias on test decisions in randomized clinical trials. Stat Med.

[51] Odgaard-Jensen J, Vist GE, Timmer A, Kunz R, Akl EA, Schünemann H (2011). Randomisation to protect against selection bias in healthcare trials. Cochrane Database Syst Rev.

[52] Tamm M, Cramer E, Kennes LN, Heussen N (2012). Influence of selection bias on the test decision. A simulation study. Methods Inf Med.

[53] Higgins JPT, Green S (2011). Cochrane Collaboration. Cochrane Handbook for Systematic Reviews of Interventions. Chichester, England.

[54] Pandis N, Polychronopoulou A, Eliades T (2011). Randomization in clinical trials in orthodontics: its significance in research design and methods to achieve it. Eur J Orthod.

[55] Herbison P, Hay-Smith J, Gillespie WJ (2011). Different methods of allocation to groups in randomized trials are associated with different levels of bias. A meta-epidemiological study. J Clin Epidemiol.

[56] Sedgwick P (2010). Allocation concealment. BMJ.

[57] Pandis N (2012). Randomization. Part 3: allocation concealment and randomization implementation. Am J Orthod Dentofac Orthop.

[58] Meinert CL. Clinical Trials Handbook: Design and Conduct. Hoboken: John Wiley & Sons, Inc: 2012. doi: 10.1002/978111842287.

[59] Clark L, Schmidt U, Tharmanathan P, Adamson J, Hewitt C, Torgerson D (2013). Allocation concealment: a methodological review. J Eval Clin Pract.

[60] Zhao W (2013). Selection bias, allocation concealment and randomization design in clinical trials. Contemp Clin Trials.

[61] Schulz KF, Grimes DA (2002). Allocation concealment in randomised trials: defending against deciphering. Lancet Lond Engl.

[62] Viera AJ, Bangdiwala SI (2007). Eliminating bias in randomized controlled trials: importance of allocation concealment and masking. Fam Med.

[63] Hróbjartsson A, Thomsen ASS, Emanuelsson F, Tendal B, Rasmussen JV, Hilden J (2014). Observer bias in randomized clinical trials with time-to-event outcomes: systematic review of trials with both blinded and non-blinded outcome assessors. Int J Epidemiol.

[64] Esdaile JM, Mackenzie T, Barré P, Danoff D, Osterland CK, Somerville P (1992). Can experienced clinicians predict the outcome of lupus nephritis?. Lupus.

[65] Funk M, Pooley-Richards RL (1994). Predicting hospital mortality in patients with acute myocardial infarction. Am J Crit Care.

[66] Groves RM, Couper MP, Presser S, Singer E, Tourangeau R, Acosta GP (2006). Experiments in Producing Nonresponse Bias. Public Opin Q.

[67] Furnham A (1986). Response bias, social desirability and dissimulation. Personal Individ Differ.

[68] Berkman ND, Santaguida PL, Viswanathan M, Morton SC. The Empirical Evidence of Bias in Trials Measuring Treatment Differences. Methods Research Report. (Prepared by the RTI-UNC Evidence-based Practice Center under Contract No. 290-2007-10056-I.) AHRQ Publication No. 14-EHC050-EF. Rockville: Agency for Healthcare Research and Quality; 2014. 25392898

[69] Djulbegovic B (2009). The paradox of equipoise: the principle that drives and limits therapeutic discoveries in clinical research. Cancer Control.

