# Supplementary material for: Mechanisms and direction of allocation bias in randomised clinical trials
Source: BMC Med Res Methodol. 2016 Oct 7;16:133. doi: 10.1186/s12874-016-0235-y (PMC5055724; doi:10.1186/s12874-016-0235-y)
Supplement: Additional file 1: — Search Methods and Sensitivity Analysis.(DOCX 44 kb) [file 12874_2016_235_MOESM1_ESM.docx]

**Appendix A – Search methods**

**Empirical studies:**

PubMed:

*(Allocation Concealment OR concealment of allocation OR Selection bias OR allocation bias]) AND (Interview OR Empirical OR questionnaire)*

Cochrane Methodology Register:

*(Selection bias OR Allocation bias OR allocation concealment) AND (Empirical OR Interview OR Questionnaire)*

Google Scholar:

We searched for the following phrases and kept the first 100 hits for each search phrase. We then removed doublets.

*“Allocation bias”*

*“Allocation bias” AND “empirical”*

*“Allocation bias” AND “interview”*

*“Allocation bias” AND “questionnaire”*

*“allocation bias” AND “allocation concealment”*

*“allocation bias” AND “allocation concealment” AND “empirical”*

*“allocation bias”AND “allocation concealment” AND “interview”*

*“allocation bias” AND “allocation concealment” AND “questionnaire”*

*“allocation bias” AND “allocation concealment” AND “mechanism”*

*“allocation bias” AND “allocation concealment” AND “survey”*

All searches were repeated using *“selection bias”* instead of *“allocation bias”*

**Figure 1: Flowchart for empirical studies:**

PubMed
[N = 2210]

Cochrane Methodology Register
[N = 145]

Google Scholar
[N = 1985]

Dublets excluded
[N = 921]

Unique publications
[N = 3419]

Publications read in full text
[N = 42]

Studies included
[N = 2]

Publications excluded based on abstract or title
[N = 3375]

Excluded full text publications [N = 40]

- Motives/methods in individual trial [N = 5]
- Individual trial, but irrelevant [N = 5]
- Other bias than allocation [N = 17]
- Not focusing on RCTs [N = 5]
- Bias in several individual studies [N = 2]
- Estimate of bias magnitude [N = 1]
- Researchers access to subjects [N = 1]
- Not empirical study [N = 3]
- Blinding, not allocation concealment [N = 1]

**Theoretical publications:**

PubMed search

*Allocation concealment OR Concealment of allocation*

Cochrane Methodology Register

*“Allocation concealment OR concealment of allocation*”

Google Scholar:

We searched for the following phrases and kept the first 100 hits for each search phrase. We then removed doublets.

*“allocation concealment”*

*“allocation concealment” AND “selection bias”*

*“allocation concealment” AND “allocation bias”*

Textbook search:

Same technique as employed in Moustgaard et. al(1).

**Figure 2: Flowchart for theoretical studies**

PubMed
[N =1475]

Cochrane Methodology Register
[N =276]

Google Scholar
[N = 300]

Dublets excluded
[N = 213]

Unique publications
[N = 1852]

Publications read in full text
[N = 130]

Studies included
[N = 53]

Publications excluded based on abstract or title
[N = 1722]

Excluded full text publications [N = 73]

- Quality assessment without mention of allocation bias [N = 22]
- No (or very little) mention of mechanisms or directions of allocation bias [N = 22]
- Assessment of reporting quality [N = 18]
- Quality assessment of trial, with no mention of allocation bias [N = 5]
- Random sequence generation [N = 3]
- Minimization [N = 2]
- RCT vs non-RCT [N = 1]

Textbooks
[N = 14]

**Appendix B - Sensitivity Analysis:**

Five trials were found that examined motives or methods of potential allocation bias in individual trials, however none of these directly asked the involved researchers about motives or methods for subversions

**Table 1:** Publications examining allocation bias in individual trials.

| **Trial ID** | **Info on study** | **Principal findings** | **Motives for subversion** | **Methods for subversion** |
| --- | --- | --- | --- | --- |
| Swingler 2000 | Looks at issues around envelope randomisation in an effectiveness trial of a diagnostic test. | 16 envelopes were improperly handled after exclusions (seven were opened, nine were lost) | Does not report on motives for potential subversions. | Some envelopes were opened for excluded patients, which could be a method of subversion. |
| Kennedy 1997 | Only abstract was available. Examines two methods of allocation; envelopes or centralised. | Concludes that use of envelopes might lead to lack of allocation concealment. | Does not report on motives for potential subversions. | Study concludes that using envelopes might mean lack of allocation concealment |
| Boyd 1997 | Examines randomisation in Canadian breast cancer screening study | Makes no conclusion as to whether subversions took place, however these were theoretically possible | Does not report on motives for potential subversions. | Subversions were possible as a coordinator knew the randomization schedule before allocation took place |
| Peto 1999 | Examines the randomization in the Captopril Prevention Project | Concludes that envelope randomisation was frequently violated, and results of study are unreliable | Does not report on motives for potential subversions. | Concludes that allocations were tampered with through the opening of envelopes before randomization |
| Jordhøy 2002 | Looks at lack of allocation concealment when using cluster randomization | Finds that suspicions of biased selection were strongly supported. | Claims that the motive of biased selection was practical considerations. | Does not report on methods of potential subversions |

**References:**

1. Moustgaard H, Bello S, Miller FG, Hróbjartsson A. Subjective and objective outcomes in randomized clinical trials: definitions differed in methods publications and were often absent from trial reports. J Clin Epidemiol. 2014 Dec;67(12):1327–34.
